# Preparation and Performance Study of Large Volume Foamed Lightweight Soil for an Intelligent Networked Vehicle Test Site

**DOI:** 10.3390/ma15155382

**Published:** 2022-08-04

**Authors:** Hao Liu, Gang Liu, Huqiang Wang, Huiwen Wan, Xiaoyang Xu, Cong Shen, Jiaqi Xuan, Qiqing He

**Affiliations:** 1 China Construction Second Engineering Bureau, East China Company Ltd., Shanghai 200135, China; 2 School of Materials Science and Engineering, Wuhan University of Technology, Wuhan 430070, China

**Keywords:** intelligent networked vehicles, test site, foamed lightweight soil, preparation, performance

## Abstract

The foamed lightweight soil (FLS) with superior performance was prepared to be used as a subgrade filling material in an intelligent networked vehicle test site. The production process of practical engineering FLS is simulated in the laboratory. The performance of the prepared FLS is the same as that of practical engineering. The test results show that the FLS prepared with 30% cement, 30% granulated blast furnace slag, and 40% fly ash as cementing material has a flow factor of 175 mm. It has good fluidity and is easy to pump. Wet density reaches 593 kg/m^3^ within the range of the control index of 600 ± 30 kg/m^3^. The compressive strength of 7 d and 28 d reaches 0.82 MPa and 1.90 MPa, respectively, which is higher than the design strength of 0.5 MPa and 1.0 MPa. Compared with FLS made of pure cement as a cementing material, the FLS has a low heat of hydration, good volume stability, no cracks on the surface, excellent sulfate resistance, good economy, and low carbon characteristics. In the meantime, it can reduce 70% CO_2_ emissions in cement production. The advanced vehicle-mounted mobile intelligent control system is flexible and convenient in actual engineering construction. It can display the wet density of FLS online, which is easy to adjust and control to ensure the quality stability of FLS.

## 1. Introduction

An intelligent connected vehicle (ICV) is the organic combination of the Internet of Vehicles and intelligent vehicles. It is equipped with advanced onboard sensors, controllers, actuators, and other devices. ICVs integrate modern communication and network technologies to realize the exchange and sharing of intelligent information between vehicles and people, roads, and backgrounds. They achieve safe, comfortable, energy-saving, efficient driving, and, ultimately, can replace the operation of a new generation of cars.

In order to ensure the safety and reliability of intelligent connected cars before they hit the road, relevant tests must be conducted on a closed test track [1]. By building nearly 100 simulated and virtual scenarios on real sites, the testing process of intelligent connected cars is accelerated, and multiple technologies, such as whole vehicles, components, communications, IoT, big data, environmental perception, and AI algorithms are tested comprehensively in order to evaluate the safety, comfort, energy consumption, efficiency, intelligence level, and much more [2].

A few developed countries have built several closed test sites for testing in the R&D phase, such as: the top ten self-driving car test sites in the United States, Asta-Zero in Sweden, K-CITY in South Korea, etc. [3]. China’s intelligent networked vehicles have been developing very rapidly recently, but the construction of supporting closed test sites has just started, and most of them have limited scenarios, which cannot meet the R&D and testing requirements of intelligent networked vehicles. Recently, a modern large-scale intelligent networked vehicle test site is being built in Wuhan, China, and the complete test system is the most advanced intelligent networked vehicle test system in China. During the construction of the test track, high demands were placed on the level of the road surface. According to the preliminary geological survey and design specifications, the roadbed of the test track needed to be filled with more than 680,000 layers of foamed lightweight soil. Pouring such a large volume of foamed lightweight soil is rare globally.

Foamed lightweight soil (FLS) is a kind of light cement-based material [4], a new type of green material with many closed pores after the foaming agent is made into foam by physical method and mixed evenly with a certain proportion of cement slurry after natural curing [5]. Compared with conventional fill, FLS has significant advantages, such as lightweight, higher fluidity [6], easy application, high strength after hardening [7], good integrity, higher durability, and low cost [8]. These advantages significantly reduce the load, soil pressure, and thickness of the shallow layer in road base treatment, thereby improving the quality of road base construction [9]. Due to the backward synthesis technology of foaming agent, the foam prepared was unstable in past years [10], which led to the poor quality of FLS and limited the engineering application of FLS. With the progress of science and technology, the foaming agent synthesis technology has improved significantly in the past 10 years, and the foam quality is stable. At the same time, there is much new equipment for preparing FLS, which significantly stabilizes and improves the construction quality of FLS. So, FLS has been applied in many engineering fields, with corresponding standards and specifications to guide production and construction [11]. Many expanded projects, such as steep sections of mountainous areas, urban underground pipelines, and pit filling projects, use FLS instead of an ordinary filling material [12]. FLS is also used for thermal insulation of industrial and civil buildings and structures, called foamed concrete (FC) [13].

It is known that FLS has a low demand for mechanical properties. Part of the granulated blast furnace slag and fly ash are usually used instead of cement in the preparation of FLS [14]. It is widely known that the granulated blast furnace slag and fly ash have good potential activity and are high-quality admixtures for preparing cement-based materials [15]. Replacing cement with some granulated blast furnace slag and fly ash can significantly reduce the hydration heat of the system, which is conducive to improving the long-term mechanical properties and durability of FLS [16]. Several studies on the effects of supplementary cementitious materials on the workability and mechanical properties of FLS indicated that adding silica fume and FA can improve the compressive strength and flow factor of FLS [17]. Yu Junyan [18], Nong Feibi [19], Tae-Hyung Kim [20], and others used FLS as a roadbed filling material and found that the settlement value of the roadbed was small, which is much less than the allowable value of the settlement. Deng Fei [21] also applied FLS for the roadbed treatment of high-speed railways, and achieved good results. The runway of Tokyo Airport in Japan was also filled with FLS. Yoichi Watabe and Takatoshi Noguchi also studied the relationship between vertical displacement and time of FLS. They concluded that FLS is an excellent filling material for airport runway roadbeds and has excellent economic benefits [22]. Studies show the significant advantages of using FLS as an alternative to conventional soil. FLS has lightweight and excellent fluidity. It is easy to be constructed, with high strength after hardening. Meanwhile, it can significantly reduce the self-weight load and the thickness of the shallow treatment of the roadbed.

The large amount of research results and engineering application experience of FLS are only limited to a small amount of fly ash or granulated blast furnace slag (about 30%), and the volume of engineering application is not large, consisting of only a few thousand cubic meters in most projects. This paper studied the composition and proportioning of FLS cementing materials and their physical properties. Meanwhile, advanced construction techniques and control processes of FLS were discussed concerning the actual engineering characteristics of large-volume FLS used as a roadbed filling material in the intelligent networked vehicle test site. The results are expected to provide reasonable technical support for the future practical engineering application of large-volume FLS.

## 2. Project Overview

**Figure 1:**
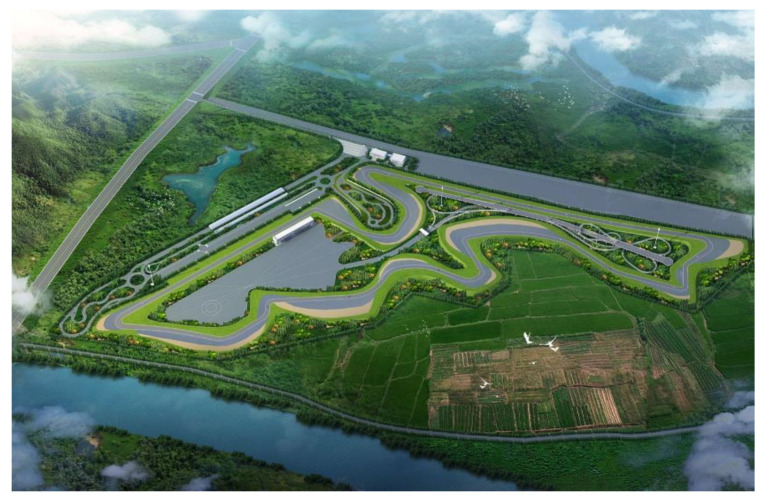
Plan view of the intelligent networked vehicle test site.

The Wuhan Intelligent Networked Vehicle Test Track is located on the banks of the Dongjing River in the Wuhan Economic and Technological Development Zone, covering an area of approximately 875,800 m^2^. As shown in Figure 1, there are 10 test areas, including an office operation and maintenance area, a high-speed and extreme performance test area, an extreme environment simulation test area, an urban traffic scenario test area, a mountain traffic scenario test area, an automatic parking test area, a rural traffic scenario test area, a multi-functional test area (virtual test plaza), a high-speed ramp simulation test area, and an extreme racing area. The test system meets the requirements of China’s standard specifications for test sites and makes full use of advanced technologies, such as big data, artificial intelligence, 5G technology, edge computing, parallel driving, etc. It will be the most technologically advanced test track in China, with the functions of the automatic driving test, ADAS test, V2X test, and limit test.

Due to the natural riverside of the track, it is a river piled plain landform feature, and the test course is about 50 m away from the Yangtze River. The test course is built in the area of a large number of vertical and horizontal ditches and ponds, with a length of about 8453.7 m, accounting for 64.3% of the main track and auxiliary roads. The ground survey measured the thickest silt layer at 22.5 m, with a maximum height difference of 6.792 m between fill and excavation. The site contains both old and new riverbed layers, with a low bearing capacity and high compressibility. It is prone to excessive and uneven settlement when loaded. In order to ensure the levelness of track, the settlement control of the foundation is extremely strict. If conventional soft clay is used as filler to fill the foundation, the settlement of soft ground will increase with the vehicle’s speed and the load on the soft ground due to its low compressive strength, which will seriously affect the safety and stability of the test track operation. Using cement combined with a gravel material for backfilling, with a high density of filling material, requires a larger size and depth of the lower pile foundation, which significantly increases the construction cost. The final design of the track foundation was to use cement fly ash gravel (CFG) piles and prefabricated PHC pipe piles, followed by graded gravel levelling, and then pouring FLS in the upper part for filling. Over 3800 boreholes were required for the entire project for CFG piles, with depths ranging from 20 to 90 m. The height of FLS ranged from 7 to 12 m, and the volume of FLS for the whole test site met 680,000 m^3^.

## 3. Materials and Methods

### 3.1. Raw Materials

The main raw materials include cement (C), fly ash (FA), granulated blast furnace slag (GBFS), and a foaming agent.

The cement is PO 42.5 cement produced by Conch Cement with a chemical composition as shown in Table 1 and physical properties as shown in Table 2. The granulated blast furnace slag is S95 grade ground slag produced by Hubei Jinshenglan Metallurgical Technology (Xianning, China). with a density of 2800 kg/m^3^, a specific surface area of 410 m^2^/kg and chemical composition as shown in Table 1. The fly ash was produced by Hubei Koneng Environmental Protection (Hanchuan Power Plant) as Class F Class II fly ash with a density of 2200 kg/m^3^ and the 15% residue of the particle on the sieve (45 µm sieving size), with the chemical composition, as shown in Table 1.

The foaming agent is a ready-mixed compound reinforcing foaming agent, model JY-SRN2, produced by Guangdong Shengrui Technology Company. The performance of the foaming agent is shown in Table 3.

### 3.2. Material Composition and Proportioning

The material composition and the combination of FLS are shown in Table 4. Group A has only cement (C); Group B has C and granulated blast furnace slag (GBFS); Group C has C and fly ash (FA); Group D has C, GBFS, and FA; and Group E has C, GBFS, and FA.

The water–cement ratio is 0.65 and the volume or mass of the foam cluster is determined according to the volume of cementitious material and water per cubic metres of FLS, the remainder being the volume of the foam cluster.

### 3.3. Technical Specifications of FLS

(1) Wet density: 600 ± 30 kg/m^3^;
(2) Foam density: 50 ± 2 kg/m^3^;
(3) Flow factor: 170 ± 10 mm;
(4) 7 d compressive strength ≥ 0.5 MPa, 28 d compressive strength ≥ 1.0 MPa.

### 3.4. Sample Preparation

(1) Foam preparation

**Figure 2:**
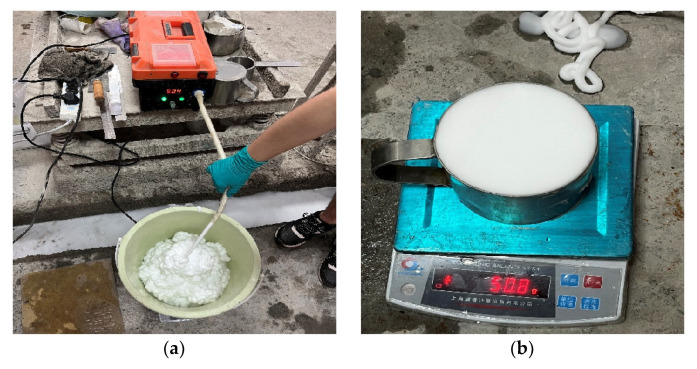

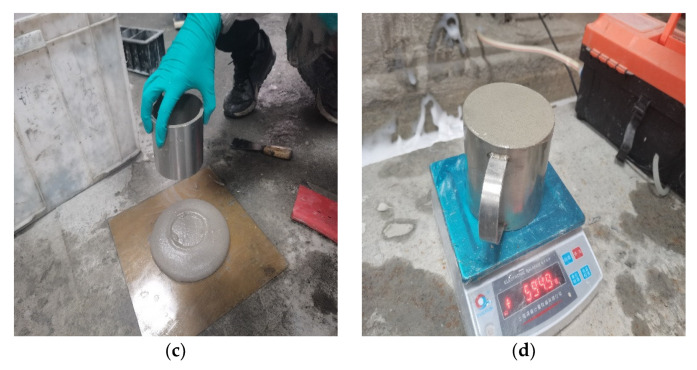
Preparation of FLS specimens: (**a**) foam preparation, (**b**) measurement of foam density, (**c**) measurement of FLS flow factor, and (**d**) measurement of FLS wet density.

Take an appropriate amount of the original foaming agent, dilute it to 100 times with water, and make physical foaming using the micro-intelligence foaming machine. The foaming theory of the micro-intelligence foaming machine is the same as that of the large compressor used in engineering. One end of the thin tube is used to inhale the diluted foaming agent, and the other end of the thick tube is used to produce relatively stable foam, as shown in Figure 2a. The foam is delicate, smooth, and uniform in size without large bubbles. Foam density can be controlled by adjusting the knob and changing the pressure of compressed air. The foam density used in the experiment is about 50 kg/m^3^, as shown in Figure 2b.

(2) Sample preparation

The required C, FA, GBFS, and water are weighed according to the designed mix ratio, and the slurry is made by mixing with a mixer for 1 min. Then, the pre-prepared foam is added and stirred for 2 min, and the uniform FLS can be prepared. Then, the flow factor (as shown in Figure 2c) and wet density (as shown in Figure 2d) of FLS are tested. If the flow factor meets the requirements of 170 ± 10 mm and wet density of 600 ± 30 kg/m^3^, the prepared FLS can be poured into a mould of 100 × 100 × 100 mm, covered with plastic film, and put it into a curing chamber with a temperature of 20 °C and humidity of 90%. After curing for 24 h, the excess FLS on the mould’s surface is scraped, and then covered with plastic film for another 24 h. When curing for 48 h, the FLS can demould and sealed with plastic bags, then placed in the standard curing room until the specified age.

### 3.5. Performance Test Method of FLS

(1) Flow factor: according to CECS 249-2008 “Technical Specification for Cast-in-place Foamed Lightweight Soil”, as shown in Figure 2c. Firstly, the stainless steel cylinder with a diameter of 80 mm and a height of 80 mm is placed on the flat glass plate or acrylic plate. Secondly, the prepared FLS is poured into the cylinder, and the excess FLS is scraped off the surface with a scraper, and then the cylinder is gently lifted. Finally, the diameter of the pulp cake on the plate is measured with calipers in two directions perpendicular to each other, and the arithmetic mean value is taken as the result of the flow factor.
(2) Wet density: according to CECS 249-2008 “Technical Specification for Cast-in-place Foamed Lightweight Soil”, as shown in Figure 2d. Clean the inner and outer walls of the 1L stainless steel capacity cylinder with a cloth, weigh it electronically, and reset it to zero. Then, pour the prepared FLS into the capacity cylinder, scrape it flat with a scraper, wipe the outer wall of the cylinder with a cloth, and weigh it. The reading on the electronic scale is the wet density of the FLS.
(3) Compressive strength: mechanical properties of FLS are tested following the GBT 11969-2020 “Test Method for the performance of Autoclaved Aerated Concrete”. First, use callipers to measure the exact size of the compression surface of 100 × 100 × 100 mm specimen, then put the specimen in the center of the testing plate, and start the testing machine. When the upper pressing plate is close to the specimen, adjust the ball seat to balance the contact. Finally, the load was uniformly added at the loading speed of 0.1 ± 0.02 kN/s until the specimen was destroyed, and the failure load P was recorded. Then, the compressive strength of FLS can be calculated.

## 4. Physical Properties of FLS

### 4.1. Characteristics of FLS Material Composition

As FLS is mainly used as a filling material for road foundations, its compressive strength requirements are not high. As shown in Table 4, the cementitious material used to prepare FLS can be pure cement or partial cement with a large amount of mineral admixture, and its performance can still meet the design requirements. Concerning the factors such as preparation cost, the internal temperature increase in the bulk FLS, volume stability, and durability, Specimen D (30% cement, 30% GBFS and 40% FA) is recommended. If we consider CO_2_ emissions to the atmosphere from the production of cement [23], based on a calculation that for every 1 t of cement produced, approximately 0.7 t of CO_2_ is emitted to the atmosphere [24], using sample A would emit approximately 241.5 kg of CO_2_, sample B or sample C would emit approximately 144.9 kg of CO_2_ [25]. In comparison, sample D would only emit 72.5 kg of CO_2_ per 1 t. It concludes that using sample D would emit 70% less CO_2_ than using sample A. Therefore, FLS prepared using specimen D meets the mechanical properties requirements and has low carbon properties.

Specimen D is a cementitious system using large doses of GBFS and FA with less cement, which has low carbon properties and belongs to green building material. GBFS is a product made from industrial waste discharged from the smelting of pig iron in blast furnaces and then ground up. FA is a powder collected from flue gases of coal-fired power plants, both of which are industrial solid waste. As both GBFS and FA have potential cementing activity, they can produce secondary hydration under the excitation of Ca(OH)_2_, the hydration product of cement, which can significantly improve the mechanical properties and durability of the system, especially the resistance to sulphate.

### 4.2. Physical Properties of FLS

**Figure 3:**
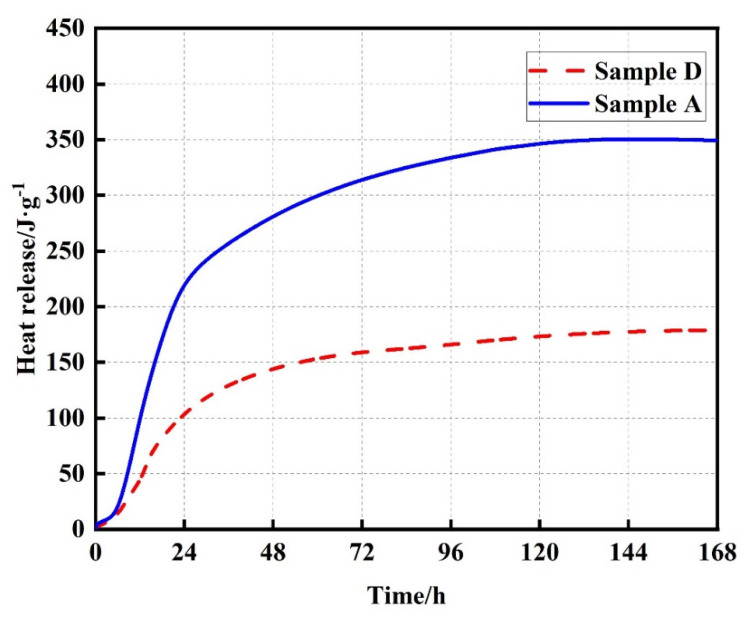
Hydration heat curves for specimen A and specimen D.

The hydration heat of sample A and sample D were measured using a Setaram cement hydration tester (Type C80). Results were shown in Figure 3. The cumulative hydration heat of pure cement sample A was 350 J/g for 7 d, while sample D was approximately 180 J/g for 7 d, almost half of sample A. This means that the internal temperature increase in FLS prepared using material composition and proportion of sample D is much lower than that of FLS prepared from sample A.

The sulphate erosion resistance test was carried out on specimen A (cement) and specimen D (30% cement, 30% GBFS, 40% FA) using GBT 749-2008 [26]. The experimental results showed that the sulphate erosion resistance coefficient of specimen A was 0.83, while the sulphate erosion resistance coefficient of specimen D was 0.95, indicating that FLS prepared using specimen D has excellent sulphate erosion resistance. This proves that FLS prepared from sample D has excellent resistance to sulphate attacks.

The properties of FLS prepared by repeated laboratory tests using the material composition and ratios in Table 4 are shown in Table 5. Table 5 indicates that the flow factor and wet density of the freshly mixed FLS, and the 7 d and 28 d compressive strength of hardened FLS meet the requirements for the designed technical specifications. In terms of production cost, FLS prepared by using the material composition and proportion of specimen D is the most economical. Therefore, the sample D solution was used in the actual project to cast FLS.

As can be seen from Table 5, under the condition of a similar wet density of FLS, the 7 d and 28 d compressive strength of sample B is significantly higher than that of sample C, due to the fact that the potential activity of granulated blast furnace slag is better than that of fly ash, but both of them are lower than that of pure cement sample A. Since most fly ash particles are spherical, some granulated blast furnace slag particles are ellipsoid, and some are irregular. The shape effect of granulated blast furnace slag is not as good as fly ash, so the flow factor of sample B is smaller than that of sample C, but both their flow factor is more significant than that of pure cement sample A because the water requirement of granulated blast furnace slag and fly ash is smaller than that of cement. Sample D thoroughly explains the combined excitation effect of granulated blast furnace slag and coal powder ash. When the cement content is only 30%, the compressive strength of 7 d and 28 d still reaches 0.82 MPa and 1.90 MPa, respectively, which is far higher than the design requirements.

**Figure 4:**
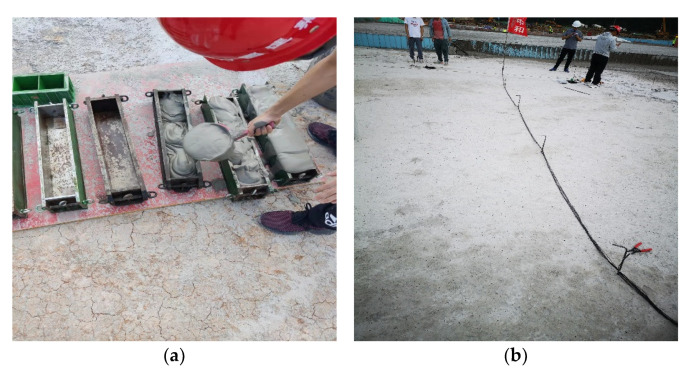
Photos of the construction site: (**a**) on-site sampling tests, and (**b**) FLS surface at the site.

The compressive strength of specimens taken on-site at the construction site was similar to that of Table 5, as shown in Figure 4a. Then, 24 h after pouring, the construction personnel can walk on the surface of FLS without any cracks arising on the surface, indicating that the volume stability of FLS is positive, as shown in Figure 4b.

## 5. Advanced Preparation Process of FLS

In practical engineering, the FLS preparation process was divided into three main parts: firstly, slurry preparation; secondly, vehicle-mounted mobile intelligent control centre; and thirdly, on-site pouring and maintenance.

### 5.1. Slurry Preparation

The storage tank for preparing the raw materials (C, GBFS, FA) can be chosen in an open, flat place within a few kilometres of the construction site so that materials can be easily transported by large bulk filling trucks. The material was measured at 30% cement, 30% GBFS and 40% FA by weight, fed into the slurry mixer and then mixed with water at a W/B ratio of 0.65 for 30 s to make a slurry of cementitious material. The slurry is then transported by pump to the silo in the mobile intelligent control centre on the construction site. The distance can be as long or as short as a pipe, up to several kilometres.

### 5.2. Vehicle-Mounted Mobile Intelligent Control Centre

**Figure 5:**
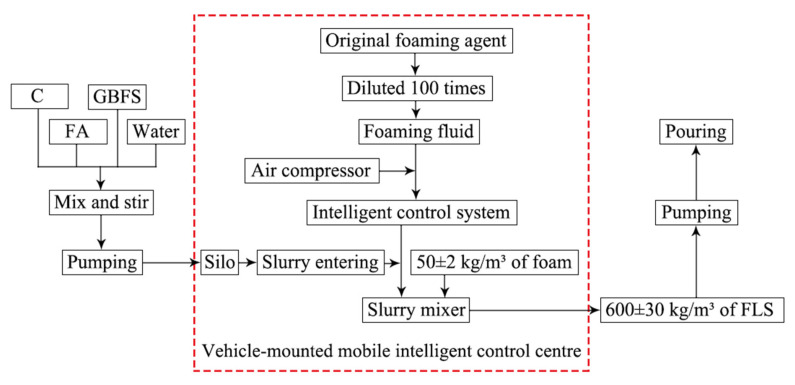
Process flow diagram for preparation of FLS.

According to the construction site conditions, the intelligent control centre was converted from a large truck and could be parked near the pouring site. The mobile intelligent control centre mainly consists of a silo, foaming liquid storage tank (100 times dilution of the original foaming agent), intelligent control system, air compressor, slurry mixer, etc. As shown in the boxed section in Figure 5.

**Figure 6:**
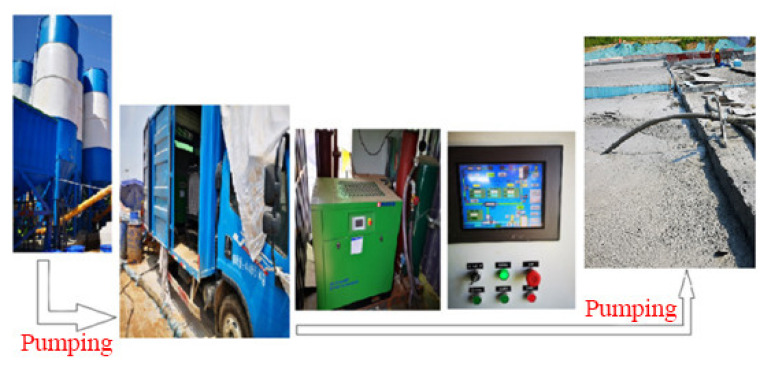
On-site process flow for FLS.

The density of foam (50 kg/m^3^) was set on the panel of the intelligent control system and was blown directly into the slurry mixer. The slurry entering the slurry mixer was adjusted according to pipeline conveying and pouring output (100 m^3^/h). The foam was mixed with slurry in the slurry mixer and measured density was displayed on the panel in the control system by the density controller. If density was in the range of 600 ± 30 kg/m^3^, FLS was made qualified. Otherwise, slurry measurement needed to be adjusted. Finally, wet density qualified FLS was pumped to the pouring site. As shown in Figure 6.

In the FLS preparation process, the vehicle-mobile intelligent control system could flexibly adjust foam density and stabilise foam quality. Secondly, wet density of FLS was displayed online at all times, convenient for adjustment and ensuring the quality of prepared FLS. Thirdly, it was conducive to pumping FLS to the pouring site over a short distance to avoid the unstable quality of FLS pouring due to the breakage of foam conveyed over a long distance.

### 5.3. On-Site Pouring and Maintenance

**Figure 7:**
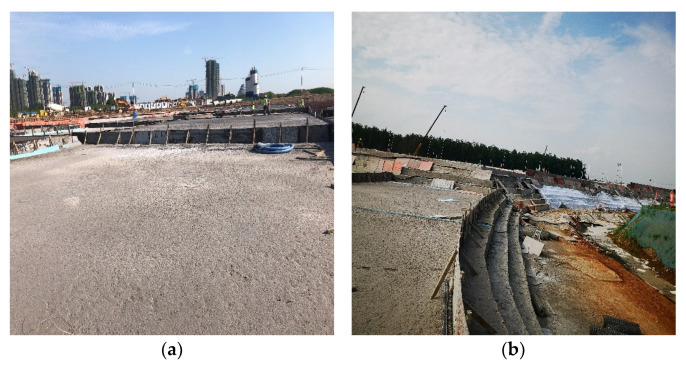
On-site pouring: (**a**) square or rectangular pouring unit and (**b**) pouring height with the wrong table at the edge of the road.

The track would be divided into square or rectangular pouring units. The maximum area of a single pouring unit should not exceed 400 m^2^, with 200 m^2^~300 m^2^ being suitable, as shown in Figure 7a. A pouring formwork surrounded each pouring unit, the height of each layer was controlled at 0.5 m, a 1.0 m wrong platform was left at the edge of the road, 2~5 mm expansion joints were left between adjacent pouring units, and the expansion joints of the upper and lower layers should be staggered as shown in Figure 7b. After each layer is poured, it needs to be maintained for 8~12 h before the second layer can be poured.

FLS did not require vibrating and were completely self-levelling. The flow factor of FLS is generally controlled at 170 ± 10 mm, with good slurry fluidity, no water secretion and collapse. The surface of FLS was hardened 8 h~10 h after pouring and can be maintained with water. After 12 h of hardening, the surface of FLS could carry an adult of 70 kg walking on it, which provides favourable conditions for the following process.

**Figure 8:**
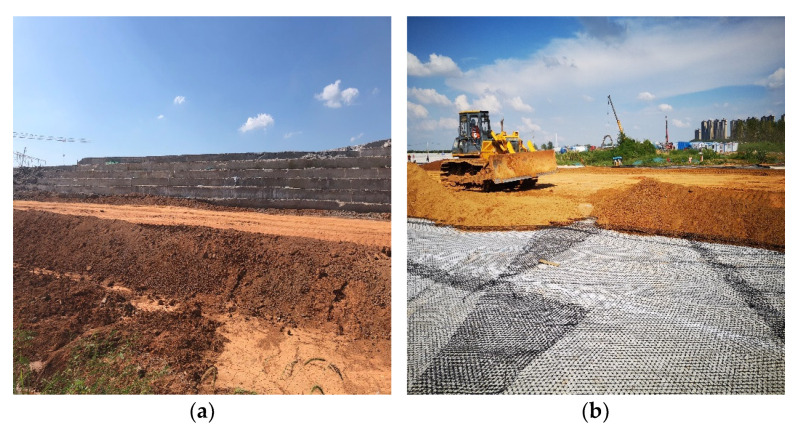
On-site maintenance: (**a**) edge compacted with cemented soil and (**b**) top layer with plastic grating and cemented soil.

After the top layer of FLS was poured, it was maintained with water at 4 h intervals until 28 d. The road edges were filled and compacted with cemented soil (approximate 8% cement content), as shown in Figure 8a. The top layer of FLS was covered with a plastic grating and then a layer of about 300 mm of cemented soil was added on top and compacted, as shown in Figure 8b. This protects the entire FLS from frost damage, carbonisation, environmental erosion agents, etc.

## 6. Conclusions

1. Granulated blast furnace slag and fly ash have excellent potential activity. In preparing FLS with a low strength level, a large amount of granulated blast furnace slag and fly ash can be used together to replace cement. Under their collective excitation, the performance of FLS is excellent.

2. FLS prepared by using 30% cement, 30% GBFS, 40% FA and W/B (water/binder ratio) 0.65 has a wet density controlled in the range of 600 ± 30 kg/m^3^ and compressive strength at 7 d, 28 d can reach 0.82 MPa, 1.90 MPa, respectively, which meets the design requirements of large road base filling materials.

3. FLS prepared with 30% cement, 30% GBFS, and 40% FA is characterised by low heat of hydration, good volumetric stability, superior sulphate resistance, and good economic and low carbon characteristics.

4. The advanced vehicle-mounted mobile intelligent control system is used for the production process to avoid being restricted by the site, which is flexible and convenient. The wet density of FLS is displayed online at all times, which is convenient for adjustment and ensures the quality of prepared FLS. Tt is conducive to pumping qualified FLS to the site for pouring over short distances, avoiding the unstable pouring quality due to foam breaking during long-distance transportation.

## Figures and Tables

**Table 1:** Chemical composition of cement, granulated blast furnace slag, and fly ash (wt%).

Material	CaO	SiO_2_	Al_2_O_3_	Fe_2_O_3_	MgO	SO_3_	K_2_O	Na_2_O	TiO_2_	LOI
Cement	60.11	20.92	5.76	3.24	1.15	2.86	0.88	0.14	0.31	4.17
Granulated Blast Furnace Slag	39.92	31.23	14.12	0.78	7.34	2.23	0.61	0.72	0.76	−0.29
Fly Ash	0.44	57.64	21.49	6.52	1.77	0.37	3.42	0.12	0.93	6.85

**Table 2:** Physical properties of PO 42.5 cement.

Density/(kg/m^3^)	Specific Surface Area/(m^2^/kg)	Stability/mm	Setting Times/min		Flexural Strength/MPa		Compressive Strength/MPa	
			Initial Setting Time	Final Setting Time	3 d	28 d	3 d	28 d
3100	340	2	170	235	5.6	8.7	28.1	50.4

**Table 3:** Physical properties of foaming agents.

No.	Item	JC/T2199-2013 “Foaming Agents for Foamed Concrete” Technical Specification Requirements	Test Results
1	Multiple of performed foam	15~30	20
2	Foam density, kg/m^3^	40~60	50
3	pH	8.5~10.5	10.2
4	Settling distance after 1 h, mm	≤70	35
5	Water secretion rate after 1 h, %	≤80	68
6	Slurry settling rate (curing for 1 d), %	≤8	3

**Table 4:** Compounding ratios for FLS (kg/m^3^).

No.	Cementitious Material Systems			Water	Foam
	C	GBFS	FA		
A	345	0	0	224	33.2
B	207	138	0	224	33.0
C	207	0	138	224	32.3
D	103.5	103.5	138	224	32.1

**Table 5:** Physical properties of FLS.

No.	A	B	C	D
Cementitious material systems	100%C	60%C + 40%GBFS	60%C + 40%FA	30%C + 30%GBFS + 40%FA
Flow factor, mm	170	175	178	175
Wet density, kg/m^3^	601	597	596	593
7 d compressive strength, MPa	1.16	0.95	0.63	0.82
28 d compressive strength, MPa	2.18	2.12	1.56	1.90

## Data Availability

The data that support the findings of this study are available from the corresponding author upon reasonable request.
